# Population Structure, Genetic Diversity, and Evolutionary History of *Kleinia neriifolia* (Asteraceae) on the Canary Islands

**DOI:** 10.3389/fpls.2017.01180

**Published:** 2017-06-30

**Authors:** Ye Sun, Carlos F. Vargas-Mendoza

**Affiliations:** ^1^Guangdong Key Laboratory for Innovative Development and Utilization of Forest Plant Germplasm, College of Forestry and Landscape Architecture, South China Agricultural UniversityGuangzhou, China; ^2^Key Laboratory of Plant Resources Conservation and Sustainable Utilization, South China Botanical Garden, Chinese Academy of SciencesGuangzhou, China; ^3^Escuela Nacional de Ciencias Biológicas-Instituto Politécnico NacionalMexico City, Mexico

**Keywords:** Canary Islands, evolutionary scenario, *Kleinia neriifolia*, population structure, simple sequence repeat

## Abstract

*Kleinia neriifolia* Haw. is an endemic species on the Canarian archipelago, this species is widespread in the coastal thicket of all the Canarian islands. In the present study, genetic diversity and population structure of *K. neriifolia* were investigated using chloroplast gene sequences and nuclear SSR (simple sequence repeat). The differentiation among island populations, the historical demography, and the underlying evolutionary scenarios of this species are further tested based on the genetic data. Chloroplast diversity reveals a strong genetic divergence between eastern islands (Gran Canaria, Fuerteventura, and Lanzarote) and western islands (EI Hierro, La Palma, La Gomera, Tenerife), this west–east genetic divergence may reflect a very beginning of speciation. The evolutionary scenario with highest posterior probabilities suggests Gran Canaria as oldest population with a westward colonization path to Tenerife, La Gomera, La Palma, and EI Hierro, and eastward dispersal path to Lanzarote through Fuerteventura. In the western islands, there is a slight decrease in the effective population size toward areas of recent colonization. However, in the eastern islands, the effective population size increase in Lanzarote relative to Gran Canaria and Fuerteventura. These results further our understanding of the evolution of widespread endemic plants within Canarian archipelago.

## Introduction

Most of the ocean islands are of volcanic origin and do not have any terrestrial life initially (Cox and Moore, 2010; Fernández-Mazuecos and Vargas, 2011). Due to spatial isolation and temporal limits, oceanic archipelagos provide ideal system to study evolutionary process involved in population differentiation and speciation (Juan et al., 2000; Fernández-Mazuecos and Vargas, 2011). The Canary Islands are an Atlantic volcanic archipelago composed of seven main islands and four islets, where house about 570 endemic plant species which represent about 40% of the native flora (Francisco-Ortega et al., 2000). The geographical origins of the Canarian endemic plants are mainly from Mediterranean (35%), Northwest African (25%), East African, South African, and New World (22%), and the rest (18%) are derived from Macaronesia (including the Canaries) which highlight a considerable role for intra-archipelago diversification (Caujape-Castells, 2011).

The widespread species on archipelago are expected to show strong genetic structure due to oceanic barriers that restrict gene flow among islands (Saro et al., 2015). In line with the expectations, the high total diversity within species and the relatively high differentiation among populations were revealed in endemic plants of the Canary Island (Francisco-Ortega et al., 2000). Although inter-island dispersal has been suggested as the main driver of diversification for plant taxa on the Canary Islands (Böhle et al., 1996; Francisco-Ortega et al., 1996; Sanmartín et al., 2008), few studies have been carried out to investigate the evolutionary history of populations in widespread endemic plants on the Canary Islands, except for some cases focused on the conservation of extremely endangered species. A Bayesian approach to phylogeny-based island biogeography suggest the three “central” island (Gran Canaria, Tenerife, and La Gomera) as the diversification and dispersal centers in the Canarian archipelago (Sanmartín et al., 2008), however, an east–west decreasing genetic diversity gradient is supported by a meta-analysis which indicate that the eastern islands (Lanzarote, Fuerteventura, and Gran Canaria) have higher allozyme diversity than that of the western islands (Tenerife, La Gomera, La Palma, and EI Hierro) (Caujape-Castells, 2011). More population genetic studies on the widespread endemic species are needed to further understand the pattern and process of intra-archipelago diversification of the Canarian endemic plants.

Most *Kleinia* species are stem-succulent that spread from the Canary Island, North Africa, and Arabian Peninsula to Madagascar and South Africa (Timonin et al., 2015). North African and Arabian stem-succulents of genus *Kleinia* are supposed to originate from South African stem succulents of this genus, or *in situ* divergence from North African hemisucculent *Kleinia* species (Timonin et al., 2015). *Kleinia neriifolia* Haw. is an endemic stem-succulent species on the Canary Islands. This species has a widespread distribution in all seven main islands, often grows together with succulent *Euphorbia* species at low elevation and constitute a typical feature of the arid and sub-arid Canary landscape (Halliday, 1986).

The combined use of nuclear and plastid markers could provide a powerful and complementary approach to investigate complex evolutionary process. In the present study, genetic diversity and population structure of *K. neriifolia* were investigated using chloroplast gene sequences and nuclear SSR (simple sequence repeat). The differentiation among island populations, the demographic history, and the underlying evolutionary scenarios of *K. neriifolia* are further tested based on the genetic data to get a clear picture of intra-archipelago diversification of this species on the Canary Islands.

## Materials and Methods

### Sample Collection, Microsatellite Genotyping, and Chloroplast Gene Sequencing

Three hundred and sixteen individuals from 14 populations (**Table 1**) of *K. neriifolia* were collected across seven islands of the Canarian archipelago during 2011 and 2012. Total genomic DNA was isolated from 50 mg of silica-dried leaf material using a modified procedure of Doyle and Doyle (1987). A washing buffer (2% PVP 40,000 MW, 100 mM Tris–HCl pH 8.0, 20 mM EDTA pH 8.0, 1.4 M NaCl, 0.2% mercaptoethanol) was used to rinse the leaf powder for 15 min at 65°C to remove most of polysaccharides, polyphenols, and secondary metabolites. All individuals were genotyped by polymerase chain reaction (PCR) with seven polymorphic microsatellite markers (S7-24, S7-40, S7-48, S7-60, S7-66, S7-73, S7-79) as described in Chen et al. (2012). Primers were labeled at 5′-end with fluorochromes, and PCR products were visualized on an ABI-377 fluorescence sequencer (Applied Biosystems, Carlsbad, CA, United States). Alleles were scored with GENEMAPPER version 1.51 (Applied Biosystems).

**Table 1:** Location, sample size, and gene diversity of the sampled populations of *Kleinia neriifolia* on the Canary Islands.

Island	Location/code	Latitude	Longitude	Individuals (sequenced)	Haplotype	*A*_R_	*H*	*F*_IS_
Lanzarote	Arrieta/L-Arr	29°08′07	13°27′47	24 (4)	H7 (3), H8 (1)	5.250	0.703	0.153
	Tias/L-Tia	28°57′17	13°30′59	24 (4)	H7 (4)	3.631	0.546	0.073
	Tinajo/L-Tin	29°03′58	13°40′43	22 (4)	H8 (4)	4.109	0.600	-0.093
Fuerteventura	Pajara/F-Paj	28°20′59	14°06′42	24 (3)	H8 (3)	5.726	0.704	0.383^∗∗^
	Vailebron/F-Vai	28°34′55	13°55′51	15 (4)	H7 (2), H8 (1), H9 (1)	3.827	0.603	0.204
Gran Canaria	Anden Verde/GC-Anv	28°01′19	15°46′36	24 (4)	H6 (1), H7 (2), H8 (1)	6.984	0.706	0.470^∗∗^
	Ingenio/GC-Ing	27°56′53	15°25′24	24 (4)	H7 (1), H8 (1), H9 (2)	7.090	0.719	0.205^∗∗^
Tenerife	Anaga/T-Ana	28°33′28	16°18′41	23 (3)	H2 (3)	6.491	0.736	0.139
	Arona/T-Aro	28°06′00	16°40′00	24 (4)	H2 (2), H4 (2)	5.425	0.696	0.205
La Gomera	Degollada/LG-Deg	28°06′02	17°10′03	20 (4)	H1 (1), H3 (2), H5 (1)	4.718	0.608	0.061
	Vallegranrey/LG-Val	28°07′14	17°18′44	20 (4)	H1 (1), H3 (3)	5.399	0.655	0.019
La Palma	de Belmaco/LP-Deb	28°43′03	17°46′10	24 (4)	H2 (4)	5.794	0.678	0.304^∗∗^
	San Antonia/LP-Saa	28°29′40	17°51′20	24 (4)	H2 (4)	5.412	0.686	0.410^∗∗^
EI Hierro	de la Pena/EH-Dlp	27°48′23	17°54′12	24 (8)	H1 (8)	6.191	0.679	0.290^∗∗^

*Departed from Hardy–Weinberg (^∗∗^*P* < 0.01).*

Chloroplast DNA sequences were obtained for three to eight individuals in each population. Three non-coding chloroplast regions (NCBI accession No. KX090948–KX091125), including trnL-trnL-trnF, petG-trnP, and petL-psbE, were amplified and sequenced with primer pairs of trnL5′^UAA^F(TabC) and trnF^GAA^(TabF) (Taberlet et al., 1991), petG and trnP (Huang et al., 2002), and petL and psbE (Shaw et al., 2007), respectively. Primer sequence information for nuclear SSRs and chloroplast gene regions were listed in Supplementary Table S1.

### Data Analysis

#### Genetic Diversity and Population Structure

Genetic diversity statistics, including alleles (*A*) revealed, allelic richness (*A*_R_) rarefied to the smallest sample size of 15 diploid individuals per population, observed heterozygosity (*H*_O_), gene diversity within population (*H*_S_), gene diversity in the total population (*H*_T_), inbreeding coefficient (*F*_IS_), and genetic differentiation among populations (*F*_ST_) were calculated at each SSR locus using FSTAT version 2.9.3 (Goudet, 2001). Hardy–Weinberg equilibrium and genotypic disequilibrium were tested with correction for multiple comparisons. Frequencies of null alleles were evaluated with program FREENA (Chapuis and Estoup, 2007), and a refined estimation of population differentiation (*F*_ST_) was obtained after excluding null alleles. *F*_ST_-outlier approach implemented in ARLEQUIN 3.0 (Excoffier et al., 2005) was used to test if microsatellites were affected by selection. Population structure was inferred with microsatellites using discriminant analysis of principal component (DAPC) which do not require the assumptions about Hardy–Weinberg equilibrium and linkage disequilibrium (Jombart et al., 2010). In this analysis, 70% of the total variance of the data was selected to be expressed by the retained axes of PCA. Genetic structure was also investigated using the Bayesian clustering algorithm implemented in the program STRUCTURE version 2.3.4 (Pritchard et al., 2000). The analyses were conducted by assuming an admixture model and correlated allele frequencies among populations. Ten replicated runs were carried out for each possible number of clusters (*K*) being tested from 1 to 14, the length of burn-in and Markov chain Monte Carlo was set up to 100,000 and 200,000 generations, respectively. The most likely *K* was selected by analyzing the second order rate of change of L(*K*) between successive *K* values (Evanno et al., 2005) using STRUCTURE HARVESTER (Earl and von Holdt, 2012). Additionally, a suboptimal value of *K* was searched to investigate more detailed structure signal by repeat analysis without the optimal and smaller values of *K* (Puppo et al., 2016). Individuals were assigned probabilistically to a cluster, or jointly to two or more clusters, if their genotypes were admixed. The genetic variation was partitioned by analysis of molecular variance (AMOVA) using the program ARLEQUIN 3.0 (Excoffier et al., 2005). Chloroplast haplotypes (Supplementary Table S2) were resolved and their relationships were inferred with a TCS network (Clement et al., 2000) implemented in POPART version 1.6 beta (Leigh and Bryant, 2015).

#### Evolutionary History Assessment

**FIGURE 1:**
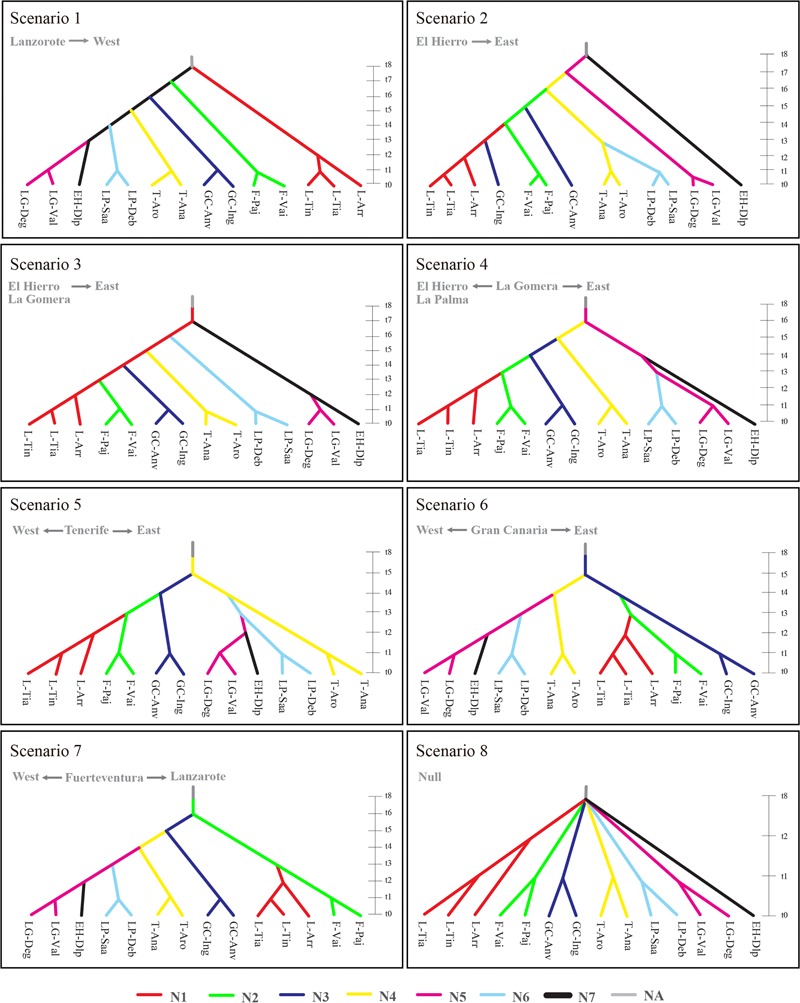
Eight evolutionary scenarios with 14 sampled populations were tested in this study, placing each island has the hypothetical ancestral population with exception of La Palma. Scenario 1: Lanzarote to west; scenario 2: El Hierro to east; scenario 3: El Hierro-La Gomera to east; scenario 4: La Gomera as oldest population; scenario 5: Tenerife as oldest population; scenario 6: Gran Canaria as oldest population; scenario 7 Fuerteventura as oldest population; finally, scenario 8 is a null scenario where all of populations split up outside of archipelago. Time scale was measured in generations. Effective population size is shown by each island (N1, Lanzarote; N2, Fuerteventura; N3, Garn Canaria; N4, Tenerife; N5, La Gomera; N6, La Palma; N7, El Hierro; NA, ancestral non-sampled population). Population codes are same as that in **Table 1**.

Based on 14 sampled populations, evolutionary scenarios of *K. neriifolia* on the Canarian archipelago were tested using both nuclear microsatellites and chloroplast sequences with DIYABC V1.0.4.46 beta (Cornuet et al., 2008, 2010). According to haplotype distribution, eight scenarios (**Figure 1**) were defined by placing each island has the hypothetical ancestral population with exception of La Palma: (1) Lanzarote to west: in this scenario, the oldest populations are in the east and youngest in the west, colonization from Lanzarote to Fuerteventura, Gran Canaria, Tenerife, La Palma, and finally to EI Hierro through La Gomera; (2) EI Hierro to east: this is the reverse scenario to the aforementioned, colonization from EI Hierro to Lanzarote, in this scenario the oldest population are in the west and youngest in the east, and there is a re-colonization of Gran Canaria from Lanzarote; (3) EI Hierro-La Gomera islands as oldest population to east: this is a variant of scenario 2, where EI Hierro and La Gomera are considered as a single ancestral population, and then a colonization eastward; (4) La Gomera as oldest population: La Gomera is the origin place, colonizing toward EI Hierro and La Palma in a westward path, and eastward to Lanzarote; (5) Tenerife as oldest population: in this case, paths of colonization are similar to the previous scenario, but the ancestral population is located in Tenerife; (6) Gran Canaria as oldest population: with a westward path to Tenerife, La Gomera, La Palma and EI Hierro, and eastward path to Lanzarote through Fuerteventura; (7) Fuerteventura as oldest population: in this scenario, one eastward path is to Lanzarote, and other westward to EI Hierro; finally, (8) null scenario: all of populations independently colonized from the outside of archipelago. In all scenarios, there is an ancestral non-sampled population with effective size of NA, we assumed one population effective size for each island NA = N1 = N2 = N3 = N4 = N5 = N6 = N7 and the priors for divergence time t8 > t7 > t6 > t5 > t4 > t3 > t2 > t1 (Supplementary Table S3). For microsatellite markers, generalized stepwise mutation model was assumed for each locus, taken a gamma distribution for individual mutation rate, as no information of mutation rate is available on this species, default values was taken (1 × 10^-5^ to 1 × 10^-2^; Cornuet et al., 2010). For chloroplast DNA sequences, Hasegawa–Kishino–Yano model (Hasegawa et al., 1985) was selected based on Akaike Information Criterion (AIC) by using the program MEGA v7.0.16 (Kumar et al., 2015) with 10% of invariant sites, taken a default uniform distribution for mean mutation rate (per site per generation) bounded between 1 × 10^-8^ and 1 × 10^-7^. Summary statistics included mean number of alleles, mean genetic diversity, mean size variance, *F*_ST_, number of haplotypes, number of segregating sites, mean of pairwise differences, and Hudson’s *F*_ST_ (Hudson et al., 1992). One million of simulations were performed for each scenario, and the 10% simulated data sets closest to observed data sets were used to estimate scenario probability and posterior distributions of parameters through a local linear regression procedure. The most likely scenario was selected by estimating their posterior probabilities using the direct estimation and logistic regression methods (Cornuet et al., 2008).

## Results

Nuclear genetic diversity and allelic richness in each population were 0.546–0.736 and 3.631–7.090 (**Table 1**), respectively. The T-Ana population possessed highest genetic diversity, and the GC-Ing population had highest allelic richness. Population F-Paj, GC-Anv, GC-Ing, LP-Deb, LP-Saa, and EH-Dlp deviated from Hardy–Weinberg equilibrium (*P* < 0.01). Number of alleles revealed per locus was 9–29, and observed heterogeneities were 0.304–0.702 (**Table 2**). No evidence was observed that the seven microsatellites were affected by selection. Excepted for the locus S7–79, inbreeding coefficient (*F*_IS_) was significantly different from 0 (*P* < 0.05) at other loci. Genetic differentiation (*F*_ST_) estimated after excluding null alleles was 0.106–0.231. AMOVA analysis showed that 16.34% of molecular variation partitioned among populations and 83.66 within populations.

**Table 2:** Characteristics of the seven nuclear microsatellites used in this study.

Locus	*A*	*A*_R_	*H*_O_	*H*_S_	*H*_T_	*F*_IS_	Null allele frequency	*F*_ST_	*F*_ST_ after excluding null alleles
S7-24	15	7.09	0.304	0.637	0.764	0.533^∗∗^	0.196	0.176	0.153
S7-40	15	6.01	0.378	0.624	0.748	0.401^∗∗^	0.156	0.180	0.163
S7-48	21	11.16	0.642	0.784	0.905	0.169^∗∗^	0.077	0.139	0.134
S7-60	29	10.65	0.583	0.731	0.866	0.209^∗∗^	0.083	0.164	0.160
S7-66	12	5.65	0.589	0.624	0.694	0.083^∗^	0.082	0.111	0.106
S7-73	18	10.01	0.702	0.760	0.876	0.076^∗∗^	0.045	0.142	0.135
S7-79	9	4.81	0.491	0.502	0.653	0.026	0.029	0.244	0.231
Overall	17	7.91	0.527	0.666	0.787	0.214^∗∗^	0.096	0.163	0.153

*Departed from Hardy–Weinberg (^∗^*P* < 0.05, ^∗∗^*P* < 0.01).*

**FIGURE 2:**
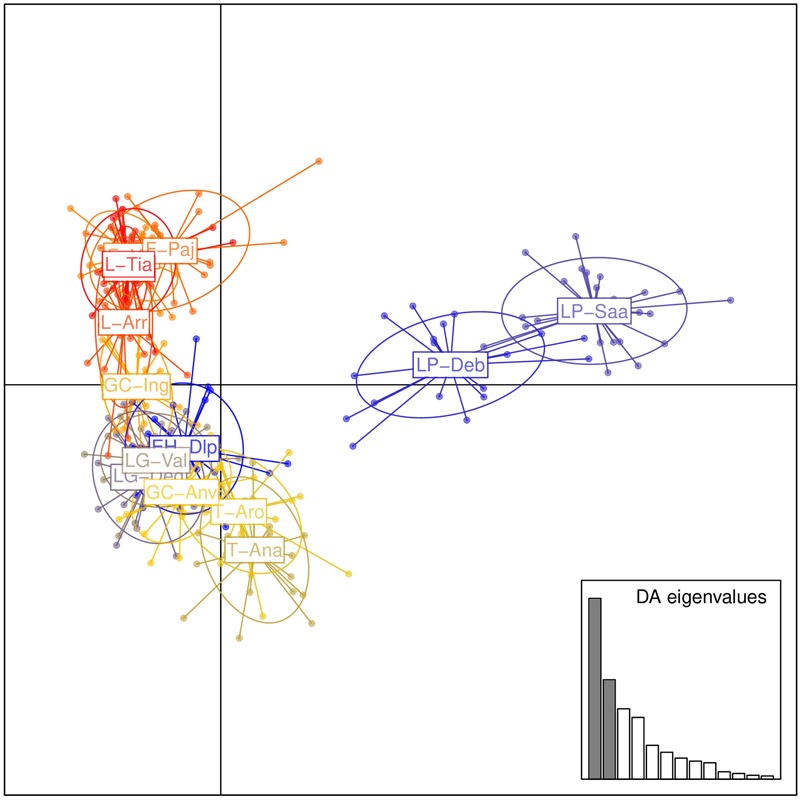
Population structure of *Kleinia neriifolia* revealed with microsatellite markers using discriminant analysis of principal component. Population codes are same as that in **Table 1**.

**FIGURE 3:**
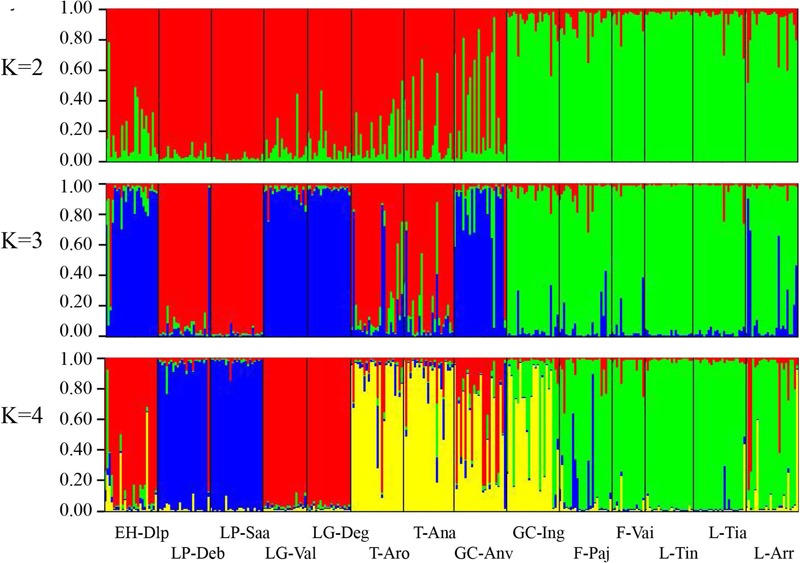
Genetic structure of *Kleinia neriifolia* revealed with microsatellite markers using STRUCTURE analyses. Individual proportion of the membership were showed when two, three, or four clusters defined by STRUCTURE analyses, respectively. Population codes are same as that in **Table 1**.

Population structure was evaluated by DAPC (**Figure 2**). The first axis distinguished populations on La Palma (LP-Saa and LP-Deb) from the others, and the second axis separated populations on eastern islands (Lanzarote, Fuerteventura, and Gran Canaria) from that on western islands (Tenerife, La Gomera, and EI Hierro) with an exception for the GC-Anv population, which showed a more close relationship to western islands than eastern islands. In STRUCTURE analyses, an optimal *K* = 2 was obtained. A western group (EH-Dlp, LP-Deb, LP-Saa, LG-Val, LG-Deg, T-Aro, T-Ana, and GC-Anv) and an eastern group (GC-Ing, F-pa, F-vai, L-Arr, L-Tia, and L-Tin) were revealed, population GC-Anv and GC-Ing on Gran Canaria was clustered separately into each group (**Figure 3**). Some individuals, especial in population T-Aro, T-Ana, and GC-Anv, showed admixture between the two groups. A suboptimal *K* = 4 was selected if STRUCTURE analyses were performed between *K* = 3 and *K* = 14. In this situation, the western group revealed above were divided into three subgroups, population LP-Deb and LP-Saa made up a distinct gene pool, population EH-Dlp, LG-Val, and LG-Deg constituted a subgroup, and population T-Aro, T-Ana, and GC-Anv formed another subgroup together with GC-Ing, which was clustered into the eastern group when *K* = 2. Population LP-Deb and LP-Saa showed close relationship with T-Aro and T-Ana if three clusters (*K* = 3) were defined by STRUCTURE analyses.

**FIGURE 4:**
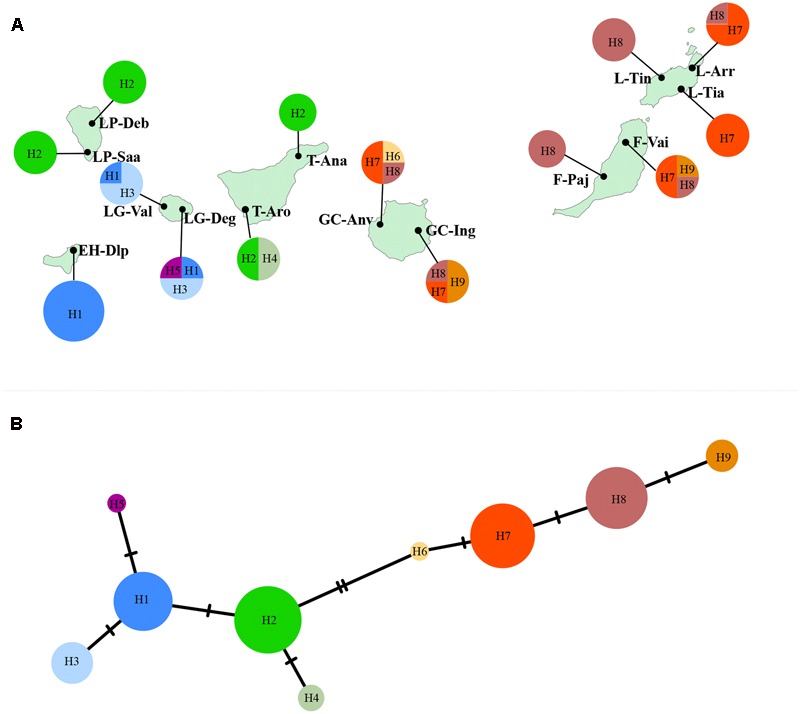
**(A)** Distributions of chloroplast haplotypes in each sampled population. Population codes are same as that in **Table 1**. **(B)** Haplotype relationships inferred with a TCS network.

DNA sequences of three non-coding chloroplast regions were combined, and a total of nine haplotypes were revealed in *K. neriifolia* (**Figure 4A**). Seven populations were fixed by a single haplotype, and population F-Vai, GC-Anv, GC-Ing, and LG-Deg possessed three haplotypes, respectively. The relationships of these chloroplast haplotypes were shown on TCS network (**Figure 4B**). Two haplogroups were distinguished and separated by two mutational steps. One haplogroup was composed by H1–H5 which were revealed only on western islands (EI Hierro, La Palma, La Gomera, and Tenerife). Another haplogroup was constituted of H6–H9 that presented only on eastern islands (Gran Canaria, Fuerteventura, and Lanzarote).

The scenario 6 (Gran Canaria as oldest population) got the highest posterior probability 0.726 [95% confidence interval (CI) = 0.512–0.940] using Logistic regression, but not with direct approach 0.166 (0.000–0.492), where scenario 2 had highest posterior probability 0.294 (0.000–0.693), and the 95% CI of both scenarios are overlapping. Since logistic regression is better estimation than direct approximation, scenario 6 was taken as the most probable scenario. Following this scenario, Gran Canaria was firstly separated from the ancestral population 205,000 (95% CI = 107,000–295,000) generations ago (Supplementary Table S4). Then, *K. neriifolia* sequentially colonized from Gran Canaria to Tenerife 62,500 (50,300–93,600) generations ago, to La Gomera 13,800 (10,100–31,200) generations, to La Palma 6,950 (5,070–9,710), and finally to El Hierro 4,200 (1,940–4,980) generations ago in westward path. In the eastward path, Fuerteventura was colonized 13,800 (10,100–31,200) generations, and Lanzarote 6,950 (5,070–9,710) generations ago. The ancestral population had an effective population size (NA) of 36,000 (95% CI = 1,060–93,600) individuals. The effective population sizes for each island were from 23,100 (5,620–70,400) on Fuerteventura to 51,300 (20,600–90,200) on Tenerife.

## Discussion

### Population Structure and Genetic Differentiation

In the present study, chloroplast diversity reveals a strong genetic divergence between eastern islands (Gran Canaria, Fuerteventura, and Lanzarote) and western islands (EI Hierro, La Palma, La Gomera, and Tenerife), which is partially congruent with the pattern of nuclear genetic diversity. This east–west genetic split has been found in other Canarian plant lineages such as *Canarina canariensis* (Mairal et al., 2015), *Phoenix canariensis* (Saro et al., 2015), *Micromeria* species (Puppo et al., 2015), and *Euphorbia* species (Sun et al., 2016).

Cryptic species represent a very important form of biodiversity (Swenson et al., 2015), and cryptic species often tend not to coexist on islands (Vodǎ et al., 2015). Significant genetic differentiation of *K. neriifolia* between eastern and western islands may reflect a very beginning of speciation. The chloroplast haplotype network indicate that *K. neriifolia* differentiate in a non-radiative way. Genetic admixture were detected in some individuals in population T-Ana and T-Aro on Tenerife and the GC-Anv population on Gran Canaria (**Figure 3**), which could be reason that the eastern and western island groups were not separated well, and GC-Anv population showed a more close relationship to the T-Aro population than to the GC-Ing population in DAPC analysis.

#### Inter-Island Dispersal within Archipelago

The levels of the mean total genetic diversity found in plants of the Canary Islands are higher than those estimated for other oceanic archipelago, which give a hint that repeatedly arrivals of plants in the early phase of inter-island colonization (Francisco-Ortega et al., 2000). Frequent inter-island dispersals could be true for *K. neriifolia* since widespread distribution of thermophilous habitats on every island makes it possible for the successful colonization of this species. Several chloroplast haplotypes distribute more than one island, such as H1 presents on EI Hierro and La Gomera, H2 on La Palma and Tenerife, H7 and H8 on Gran Canaria, Fuerteventura, and Lanzarote. These common chloroplast haplotypes shared among islands highlight seed dispersal among islands given that the chloroplast genome is transmitted maternally in most angiosperms (Petit and Vendramin, 2007).

Plants on oceanic islands often originate from self-compatible colonizers which could establish a sexually reproducing population and get seeds without specialized pollinators (Baker, 1955; Crawford et al., 2010). The close relatives of *Kleinia*, groundsels, are apparent self-pollination with occasional outbreeding depending on season of flowering (Haskell, 1953). There is no data available about the model of pollination and breeding system for *Kleinia* species, however, high levels of inbreeding in some populations (**Table 1**) may indicate some degree of selfing. Island populations are expected to show increased inbreeding relative to mainland populations due to smaller population size (Frankham, 1998), this may be the reason why some populations in our study deviated from Hardy–Weinberg equilibrium. The fruits of *K. neriifolia* have conspicuous feathery pappus that enables the seeds to be carried by the wind. Moreover, inter-island dispersals could occur since stochastic and non-standard mechanisms dominate long-distance dispersal in plants (Higgins et al., 2003). The relatively high reproductive capacity, short generation time, and efficient dispersal mechanism of the members in family Asteraceae facilitate their colonization, thus it is not surprised that this family of flowering plants have the highest number of oceanic endemic species (Crawford et al., 2010).

#### Historical Demography and Evolutionary Scenario of *K. neriifolia*

There are no consistent signals of demographical change observed for plant species widespread on the Canary Islands. For non-endemic species, a genetic bottleneck after colonization were revealed in widespread annual *Scrophularia arguta* (Valtueña et al., 2016), however, *Cistus monspeliensis* showed higher genetic variation among oceanic island populations than those of the continent (Fernández-Mazuecos and Vargas, 2011). A moderate signal of population expansion in the central islands (Gran Canaria, Tenerife, and La Gomera) was detected in endemic palm species *P. canariensis* (Saro et al., 2015). In our study, the effective population size show contrast trend in western and eastern island groups. In the western islands, there is a slight decrease in the effective population size toward areas of recent colonization. Despite much older than EI Hierro or La Palma, La Gomera show lower nuclear allelic richness and genetic diversity relative to the other islands. Thus, it is possible that Tenerife between La Gomera and Gran Canaria was necessary for successful plant colonization (Caujape-Castells, 2011), and this explain why populations on Tenerife show some levels of genetic admixture with Gran Canaria (**Figure 3**). In the eastern islands, the effective population size increase in Lanzarote relative to Gran Canaria and Fuerteventura. Gran Canaria possess highest number of chloroplast haplotypes and highest nuclear allelic richness relative to the other islands, which is consistent with the DIYABC results that Gran Canaria is the origin place of *K. neriifolia*. Population expansions within Lanzarote could be result from recurrent cycles of extinction and re-colonization (Brown and Pestano, 1998; Navascués et al., 2006). The predominance of westerlies during the Quaternary, potentially enhanced dispersal from Gran Canaria to Fuerteventura and Lanzarote (Caujape-Castells, 2011).

*Kleinia neriifolia* often grows together with succulent *Euphorbia* species and constitute the thermophilous and xerophilous vegetation on the Canary Islands. In a recent study, Tenerife-La Gomera are suggested as the origin place of the succulent *Euphorbia* species (sect. Aphyllis subsect. Macaronesicae) on the Canary Islands (Sun et al., 2016). Three palaeo-islands of Tenerife and La Gomera are rather old and formed a small archipelago until the early Quaternary (Trusty et al., 2005). Considering Gran Canaria as the origin place of *K. neriifolia*, it thus argue for an extension of the thermophilous and xerophilous vegetation from the “central” islands (Gran Canaria, Tenerife, and La Gomera) to the east and west, this fit the biogeographic prediction that central islands are the predominant diversification and dispersal centers in the Canarian archipelago (Sanmartín et al., 2008).

Exceptionally high diversification rate driven by the onset of the Mediterranean climate has been revealed in some genera of family Asteraceae on the Canarian archipelago, such as *Argyranthemum* and *Cheirolophus* (Francisco-Ortega et al., 1996; Fiz-Palacios and Valcárcel, 2013; Vitales et al., 2014). But, *K. neriifolia* is the only native species of *Kleinia* on the Canary Islands. The low diversification rate of *Kleinia* on the Canary Islands could be mainly due to its very young evolutionary times, and differentiation might be impeded by gene flow (Herben et al., 2005) given some levels of genetic admixture revealed in T-Ana, T-Aro, and GC-Anv population.

## Author Contributions

YS conceived and designed the experiments, performed the experiments, analyzed the data, and wrote the paper. CV-M analyzed the data and reviewed drafts of the paper.

## Conflict of Interest Statement

The authors declare that the research was conducted in the absence of any commercial or financial relationships that could be construed as a potential conflict of interest.
