# Supplementary material for: Population Structure, Genetic Diversity, and Evolutionary History of Kleinia neriifolia (Asteraceae) on the Canary Islands
Source: Front Plant Sci. 2017 Jun 30;8:1180. doi: 10.3389/fpls.2017.01180 (PMC5492869; doi:10.3389/fpls.2017.01180)
Supplement: Supplementary file 1 [file Table_1.DOCX]

Table S1. Primer sequences for amplification of nuclear SSRs and chloroplast gene regions used in the present study.

| Nuclear SSR Locus | Primer sequence (5’-3’) | Reference |
| --- | --- | --- |
| S7-24 | F: GAGCTCTAGAAGGGGCAGGT  R: CACCCCTTTTTCCTACTCCA | Chen, J. L., Li, C., Sun, Y., Wang, F. G., Xing, F. W. (2012). Microsatellite markers for Kleinia neriifolia, an endemic Asteraceae species on the Canary Islands. Am. J. Bot. 99, e474-e476. doi: 10.3732/ajb.1200166 |
| S7-40 | F: AATGTGTTGAATGGGAAGACG  R: TCAAGGGGTGAATTGTTCCT |  |
| S7-48 | F: CCTCCACCTTGAAAAGAATC  R: ATCGACCCCTTGGTTAGGAG |  |
| S7-60 | F: GGAATGAGAGGTGAGGGACT  R: TGTTGGTGCCTTCAAAATCA |  |
| S7-66 | F: CCCGAAGGTTTTTACCCTGT  R: CTCACCCCTTATTGCAAACAT |  |
| S7-73 | F: GAATCTTTTGGACCGGATGA  R: CACATCAGAATGTGTGTGC |  |
| S7-79 | F: CAAGGATGTGTTTCGGGTCT  R: CCGCAGTCATTGTCAGATCA |  |
| Chloroplast region | Primer sequence (5’-3’) | Reference |
| trnL-trnL-trnF | trnL5’UAAF(TabC): CGAAATCGGTAGACGCTACG  trnFGAA(TabF): ATI'TGAACTGGTGACACGAG | Taberlet, P., Gielly, L., Pautou, G., Bouvet, J. (1991). Universal primers for amplification of three non-coding regions of chloroplast DNA. Plant Mol. Biol. 17, 1105-1109. doi: 10.1007/BF00037152 |
| petG-trnP | petG: GGTCTAATTCCTATAACTTTGGC  trnP: GGGATGTGGCGCAGCTTGG | Huang, S. S., Hwang, S. Y., Lin, T. P. (2002). Spatial pattern of chloroplast DNA variation of Cyclobalanopsis glauca in Taiwan and East Asia. Mol. Ecol. 11, 2349-2358. doi: 10.1046/j.1365-294X.2002.01624.x |
| petL-psbE | petL: AGTAGAAAACCGAAATAACTAGTTA  psbE: TATCGAATACTGGTAATAATATCAGC | Shaw, J., Lickey, E. B., Schilling, E. E., Small, R. L. (2007). Comparison of whole chloroplast genome sequences to choose noncoding regions for phylogenetic studies in angiosperms: the tortoise and the hare Ⅲ. Am. J. Bot. 94, 275-288. doi: 10.3732/ajb.94.3.275 |
