# Supplementary material for: Population Structure, Genetic Diversity, and Evolutionary History of Kleinia neriifolia (Asteraceae) on the Canary Islands
Source: Front Plant Sci. 2017 Jun 30;8:1180. doi: 10.3389/fpls.2017.01180 (PMC5492869; doi:10.3389/fpls.2017.01180)
Supplement: Supplementary file 2 [file Table_2.DOCX]

Table S2. DNA sequences of the nine chloroplast haplotypes revealed in the present study.

| chloroplast haplotype | DNA sequences |
| --- | --- |
| H1 | AACATGCTTCTATGTCAATTGAAAGGGTATTACAAAGTCTCATGCAGGTCCTATAATTTG  TCAAAAGAACAACTTTGTAAGTTTCAGTCTGAATAAAAAAAATATCATTCAAATGGGGAT  TCCTTGCTCAAAGATGTTCATTTGTACATATATTATAT-TCCATTGTGATGTGATAACAG  AACAGTATTTCCGTTCAGATCCATTTGTATAAAGAGTAGTGAATGAGAAAGATAAGGAGT  TTTGAACCACTAACGAAAAAGGATAAATCAAAAGGATACGATAAATAATTAGGGAGTCAA  ATGGTCTTTTTGGGGATAGAGGGACTTGAACCCTCACGATTTTTGAAATCGACGGATTTT  CCTCTTACTATAAATTTCATTGTTGCCGGTATTGACATGTAGAACGGGACTCTATCTTTA  TTCTCGTCCAACAGTTCTTCAAAAGATCTATCAGATTATGGAGTGAATGGTTTGATCAAT  GGATATTCGATTCTTTCTTCAATATGGAATCGATTCATACCAATTCTTCTGTATTATGTA  TACAGGTTTATCCTTCAGCTTTTCTGAAGTTTCGATAGAAGGATTTCTCTACCAATGTAA  GACAATCAACTCCATTCGTTAGAACAACTTCCATCGAGTCTCTGCACCTATCCTTTTTTC  TTTTCGCTTTCTGAACCTTTGTTTGTTTTCATACAGGCGCGGCGATCAGTTGGACCTTTG  ATTAATTAACATCTCTTTTTTTG------ATTGACCTCCTCCTTTCTTTAATATCCAGGA  GGTCAAATTAAGATTGCTGTTCCAGTTAGTGTTTCAGTCGAATTCGATCGAAGAAGATCC  GAATCACGCTCTGTAGGATTTGAACCTACGACATCGGGTTTTGGAGACCCACGTTCTACC  GAACTGAACTAAGAGCGCTTTCTTATCATAAAAGATAAGATAAGACTGTAAAGAAAAGGA  TTGGCCCCAATACATCTTGTATGCATACACTATACAATATCGTAAGAATCGAAGATGTAA  GTTCTATATGATATGTCCAATGCGAATTGATCTCAAAAAATCCCTTGTTACTGCTCAAAG  GAGCAGTAATAAGTAGGGATGACAGGATTTGAACCCGTGACATTTTGTACCCAAAACAAA  CGCGCTACCAAGCCAGACATGCTGAGCTCCACATATTCTTGTACAGTCAAAAGGGGATCG  ATTCCGTAAAAGATTGGATCAGTAAATGGAAATTAACTGAAAAAATCTTTGTGAGATCGT  CAATATTGTACCGAGGGCGTCTTTAGAGTATACCGAATCAGTATAGCTATCCTTCTTCTG  ACACAGCAACGCACTTTCAATCAGTATTGGAAGGAGATGCTAAATAATTTATTTCTTCCT  TTACTTGTTGTTTGTCGATGTAAAATAATGTCGCATTCAATAGAAAATTTTAAAATTTTT  AAAAATCTATTTCTCGTTATTATGAGTTTAGGGCTAGAAACGGAGGATCAGGTAAAATCT  GAATCTGATAAGCAAGTTTCTAATAATTCATGAAATTTATTAGAATGCTCCCACAATTGT  AAGTAGATGTGAGATCTATAAATCTTTGTTATTCTATAAATCTTTGTTATTCATAGTTGT  AGAAGCACTTTTTGTTGGAATCTTTTTTT-CTTTTAAGGAATTGGTTAGTCGTCCAGTAA  CAAGTAAGAATAGTAAATTTTATTTTATAAACG------AAAAAGAACAAAGA-TCAAAC  AAATAATTGGAATCACTAATGCATGCACTGTTGTATTAAATCGAAATCTGAAGGTTCTTT  CTTGACCTAAAATGAAAGTAAGGGTTTTTTGTTCGCTGATAAAAAAAAGAATAATTTTAG  AATTTGATTACATAATGATTCAAAAAGAGTTTTATTTTAAAATGAAATTACACGACCCAG  TTCTTCTTATTCGTTTATAAGTTGAGTTGAAAAATGATGCAAGAATGCATTCGCTGATTG  CAAACGCGGTATGCGTAGATGTTACAGATGATGAATCCATTTTATACAGTTGTGACTTTA  TCCTTGTTAGTGCTGTCTATAATGATATATGAATCAATTTAATTGGTATTTTTTTATCCA  ATTTTGCAAAAAAGGAAACTCAGGTAAGTGCTTTTT-ATAAACATATGTATAAAAAGAAC |
| H2 | AACATGCTTCTATGTCAATTGAAAGGGTATTACAAAGTCTCATGCAGGTCCTATAATTTG  TCAAAAGAACAACTTTGTAAGTTTCAGTCTGAATAAAAAAAATATCATTCAAATGGGGAT  TCCTTGCTCAAAGATGTTCATTTGTACATATATTATAT-TCCATTGTGATGTGATAACAG  AACAGTATTTCCGTTCAGATCCATTTGTATAAAGAGTAGTGAATGAGAAAGATAAGGAGT  TTTGAACCACTAACGAAAAAGGATAAATCAAAAGGATACGATAAATAATTAGGGAGTCAA  ATGGTCTTTTTGGGGATAGAGGGACTTGAACCCTCACGATTTTTGAAATCGACGGATTTT  CCTCTTACTATAAATTTCATTGTTGCCGGTATTGACATGTAGAACGGGACTCTATCTTTA  TTCTCGTCCAACAGTTCTTCAAAAGATCTATCAGATTATGGAGTGAATGGTTTGATCAAT  GGATATTCGATTCTTTCTTCAATATGGAATCGATTCATACCAATTCTTCTGTATTATGTA  TACAGGTTTATCCTTCAGCTTTTCTGAAGTTTCGATAGAAGGATTTCTCTACCAATGTAA  GACAATCAACTCCATTCGTTAGAACAACTTCCATCGAGTCTCTGCACCTATCCTTTTTTC  TTTTCGCTTTCTGAACCTTTGTTTGTTTTCATACAGGCGCGGCGATCAGTTGGACCTTTG  ATTAATTAACATCTCTTTTTTTG------ATTGACCTCCTCCTTTCTTTAATATCCAGGA  GGTCAAATTAAGATTGCTGTTCCAGTTAGTGTTTCAGTCGAATTCGATCGAAGAAGATCC  GAATCACGCTCTGTAGGATTTGAACCTACGACATCGGGTTTTGGAGACCCACGTTCTACC  GAACTGAACTAAGAGCGCTTTCTTATCATAAAAGATAAGATAAGACTGTAAAGAAAAGGA  TTGGCCCCAATACATCTTGTATGCATACACTATACAATATCGTAAGAATCGAAGATGTAA  GTTCTATATGATATGTCCAATGCGAATTGATCTCAAAAAATCCCTTGTTACTGCTCAAAG  GAGCAGTAATAAGTAGGGATGACAGGATTTGAACCCGTGACATTTTGTACCCAAAACAAA  CGCGCTACCAAGCCAGACATGCTGAGCTCCACATATTCTTGTACAGTCAAAAGGGGATCG  ATTCCGTAAAAGATTGGATCAGTAAATGGAAATTAACTGAAAAAATCTTTGTGAGATCGT  CAATATTGTACCGAGGGCGTCTTTAGAGTATACCGAATCAGTATAGCTATCCTTCTTCTG  ACACAGCAACGCACTTTCAATCAGTATTGGAAGGAGATGCTAAATAATTTATTTCTTCCT  TTACTTGTTGTTTGTCGATGTAAAATAATGTCGCATTCAATAGAAAATTTTAAAATTTTT  AAAAATATATTTCTCGTTATTATGAGTTTAGGGCTAGAAACGGAGGATCAGGTAAAATCT  GAATCTGATAAGCAAGTTTCTAATAATTCATGAAATTTATTAGAATGCTCCCACAATTGT  AAGTAGATGTGAGATCTATAAATCTTTGTTATTCTATAAATCTTTGTTATTCATAGTTGT  AGAAGCACTTTTTGTTGGAATCTTTTTTT-CTTTTAAGGAATTGGTTAGTCGTCCAGTAA  CAAGTAAGAATAGTAAATTTTATTTTATAAACG------AAAAAGAACAAAGA-TCAAAC  AAATAATTGGAATCACTAATGCATGCACTGTTGTATTAAATCGAAATCTGAAGGTTCTTT  CTTGACCTAAAATGAAAGTAAGGGTTTTTTGTTCGCTGATAAAAAAAAGAATAATTTTAG  AATTTGATTACATAATGATTCAAAAAGAGTTTTATTTTAAAATGAAATTACACGACCCAG  TTCTTCTTATTCGTTTATAAGTTGAGTTGAAAAATGATGCAAGAATGCATTCGCTGATTG  CAAACGCGGTATGCGTAGATGTTACAGATGATGAATCCATTTTATACAGTTGTGACTTTA  TCCTTGTTAGTGCTGTCTATAATGATATATGAATCAATTTAATTGGTATTTTTTTATCCA  ATTTTGCAAAAAAGGAAACTCAGGTAAGTGCTTTTT-ATAAACATATGTATAAAAAGAAC |
| H3 | AACATGCTTCTATGTCAATTGAAAGGGTATTACAAAGTCTCATGCAGGTCCTATAATTTG  TCAAAAGAACAACTTTGTAAGTTTCAGTCTGAATAAAAAAAATATCATTCAAATGGGGAT  TCCTTGCTCAAAGATGTTCATTTGTACATATATTATAT-TCCATTGTGATGTGATAACAG  AACAGTATTTCCGTTCAGATCCATTTGTATAAAGAGTAGTGAATGAGAAAGATAAGGAGT  TTTGAACCACTAACGAAAAAGGATAAATCAAAAGGATACGATAAATAATTAGGGAGTCAA  ATGGTCTTTTTGGGGATAGAGGGACTTGAACCCTCACGATTTTTGAAATCGACGGATTTT  CCTCTTACTATAAATTTCATTGTTGCCGGTATTGACATGTAGAACGGGACTCTATCTTTA  TTCTCGTCCAACAGTTCTTCAAAAGATCTATCAGATTATGGAGTGAATGGTTTGATCAAT  GGATATTCGATTCTTTCTTCAATATGGAATCGATTCATACCAATTCTTCTGTATTATGTA  TACAGGTTTATCCTTCAGCTTTTCTGAAGTTTCGATAGAAGGATTTCTCTACCAATGTAA  GACAATCAACTCCATTCGTTAGAACAACTTCCATCGAGTCTCTGCACCTATCCTTTTTTC  TTTTCGCTTTCTGAACCTTTGTTTGTTTTCATACAGGCGCGGCGATCAGTTGGACCTTTG  ATTAATTAACATCTCTTTTTTTG------ATTGACCTCCTCCTTTCTTTAATATCCAGGA  GGTCAAATTAAGATTGCTGTTCCAGTTAGTGTTTCAGTCGAATTCGATCGAAGAAGATCC  GAATCACGCTCTGTAGGATTTGAACCTACGACATCGGGTTTTGGAGACCCACGTTCTACC  GAACTGAACTAAGAGCGCTTTCTTATCATAAAAGATAAGATAAGACTGTAAAGAAAAGGA  TTGGCCCCAATACATCTTGTATGCATACACTATACAATATCGTAAGAATCGAAGATGTAA  GTTCTATATGATATGTCCAATGCGAATTGATCTCAAAAAATCCCTTGTTACTGCTCAAAG  GAGCAGTAATAAGTAGGGATGACAGGATTTGAACCCGTGACATTTTGTACCCAAAACAAA  CGCGCTACCAAGCCAGACATGCTGAGCTCCACATATTCTTGTACAGTCAAAAGGGGATCG  ATTCCGTAAAAGATTGGATCAGTAAATGGAAATTAACTGAAAAAATCTTTGTGAGATCGT  CAATATTGTACCGAGGGCGTCTTTAGAGTATACCGAATCAGTATAGCTATCCTTCTTCTG  ACACAGCAACGCACTTTCAATCAGTATTGGAAGGAGATGCTAAATAATTTATTTCTTCCT  TTACTTGTTGTTTGTCGATGTAAAATAATGTCGCATTCAATAGAAAATTTTAAAATTTTT  AAAAATCTATTTCTCGTTATTATGAGTTTAGGGCTAGAAACGGAGGATCAGGTAAAATCT  GAATCTGATAAGCAAGTTTCTAATAATTCATGAAATTTATTAGAATGCTCCCACAATTGT  AAGTAGATGTGAGATCTATAAATCTTTGTTATTCTATAAATCTTTGTTATTCATAGTTGT  AGAAGCACTTTTTGTTGGAATCTTTTTTT-CTTTTAAGGAATTGGTTAGTCGTCCAGTAA  CAAGTAAGAATAGTCAATTTTATTTTATAAACG------AAAAAGAACAAAGA-TCAAAC  AAATAATTGGAATCACTAATGCATGCACTGTTGTATTAAATCGAAATCTGAAGGTTCTTT  CTTGACCTAAAATGAAAGTAAGGGTTTTTTGTTCGCTGATAAAAAAAAGAATAATTTTAG  AATTTGATTACATAATGATTCAAAAAGAGTTTTATTTTAAAATGAAATTACACGACCCAG  TTCTTCTTATTCGTTTATAAGTTGAGTTGAAAAATGATGCAAGAATGCATTCGCTGATTG  CAAACGCGGTATGCGTAGATGTTACAGATGATGAATCCATTTTATACAGTTGTGACTTTA  TCCTTGTTAGTGCTGTCTATAATGATATATGAATCAATTTAATTGGTATTTTTTTATCCA  ATTTTGCAAAAAAGGAAACTCAGGTAAGTGCTTTTT-ATAAACATATGTATAAAAAGAAC |
| H4 | AACATGCTTCTATGTCAATTGAAAGGGTATTACAAAGTCTCATGCAGGTCCTATAATTTG  TCAAAAGAACAACTTTGTAAGTTTCAGTCTGAATAAAAAAAATATCATTCAAATGGGGAT  TCCTTGCTCAAAGATGTTCATTTGTACATATATTATAT-TCCATTGTGATGTGATAACAG  AACAGTATTTCCGTTCAGATCCATTTGTATAAAGAGTAGTGAATGAGAAAGATAAGGAGT  TTTGAACCACTAACGAAAAAGGATAAATCAAAAGGATACGATAAATAATTAGGGAGTCAA  ATGGTCTTTTTGGGGATAGAGGGACTTGAACCCTCACGATTTTTGAAATCGACGGATTTT  CCTCTTACTATAAATTTCATTGTTGCCGGTATTGACATGTAGAACGGGACTCTATCTTTA  TTCTCGTCCAACAGTTCTTCAAAAGATCTATCAGATTATGGAGTGAATGGTTTGATCAAT  GGATATTCGATTCTTTCTTCAATATGGAATCGATTCATACCAATTCTTCTGTATTATGTA  TACAGGTTTATCCTTCAGCTTTTCTGAAGTTTCGATAGAAGGATTTCTCTACCAATGTAA  GACAATCAACTCCATTCGTTAGAACAACTTCCATCGAGTCTCTGCACCTATCCTTTTTTC  TTTTCGCTTTCTGAACCTTTGTTTGTTTTCATACAGGCGCGGCGATCAGTTGGACCTTTG  ATTAATTAACATCTCTTTTTTTG------ATTGACCTCCTCCTTTCTTTAATATCCAGGA  GGTCAAATTAAGATTGCTGTTCCAGTTAGTGTTTCAGTCGAATTCGATCGAAGAAGATCC  GAATCACGCTCTGTAGGATTTGAACCTACGACATCGGGTTTTGGAGACCCACGTTCTACC  GAACTGAACTAAGAGCGCTTTCTTATCATAAAAGATAAGATAAGACTGTAAAGAAAAGGA  TTGGCCCCAATACATCTTGTATGCATACACTATACAATATCGTAAGAATCGAAGATGTAA  GTTCTATATGATATGTCCAATGCGAATTGATCTCAAAAAATCCCTTGTTACTGTTCAAAG  GAGCAGTAATAAGTAGGGATGACAGGATTTGAACCCGTGACATTTTGTACCCAAAACAAA  CGCGCTACCAAGCCAGACATGCTGAGCTCCACATATTCTTGTACAGTCAAAAGGGGATCG  ATTCCGTAAAAGATTGGATCAGTAAATGGAAATTAACTGAAAAAATCTTTGTGAGATCGT  CAATATTGTACCGAGGGCGTCTTTAGAGTATACCGAATCAGTATAGCTATCCTTCTTCTG  ACACAGCAACGCACTTTCAATCAGTATTGGAAGGAGATGCTAAATAATTTATTTCTTCCT  TTACTTGTTGTTTGTCGATGTAAAATAATGTCGCATTCAATAGAAAATTTTAAAATTTTT  AAAAATATATTTCTCGTTATTATGAGTTTAGGGCTAGAAACGGAGGATCAGGTAAAATCT  GAATCTGATAAGCAAGTTTCTAATAATTCATGAAATTTATTAGAATGCTCCCACAATTGT  AAGTAGATGTGAGATCTATAAATCTTTGTTATTCTATAAATCTTTGTTATTCATAGTTGT  AGAAGCACTTTTTGTTGGAATCTTTTTTT-CTTTTAAGGAATTGGTTAGTCGTCCAGTAA  CAAGTAAGAATAGTAAATTTTATTTTATAAACG------AAAAAGAACAAAGA-TCAAAC  AAATAATTGGAATCACTAATGCATGCACTGTTGTATTAAATCGAAATCTGAAGGTTCTTT  CTTGACCTAAAATGAAAGTAAGGGTTTTTTGTTCGCTGATAAAAAAAAGAATAATTTTAG  AATTTGATTACATAATGATTCAAAAAGAGTTTTATTTTAAAATGAAATTACACGACCCAG  TTCTTCTTATTCGTTTATAAGTTGAGTTGAAAAATGATGCAAGAATGCATTCGCTGATTG  CAAACGCGGTATGCGTAGATGTTACAGATGATGAATCCATTTTATACAGTTGTGACTTTA  TCCTTGTTAGTGCTGTCTATAATGATATATGAATCAATTTAATTGGTATTTTTTTATCCA  ATTTTGCAAAAAAGGAAACTCAGGTAAGTGCTTTTT-ATAAACATATGTATAAAAAGAAC |
| H5 | AACATGCTTCTATGTCAATTGAAAGGGTATTACAAAGTCTCATGCAGGTCCTATAATTTG  TCAAAAGAACAACTTTGTAAGTTTCAGTCTGAATAAAAAAAATATCATTCAAATGGGGAT  TCCTTGCTCAAAGATGTTCATTTGTACATATATTATAT-TCCATTGTGATGTGATAACAG  AACAGTATTTCCGTTCAGATCCATTTGTATAAAGAGTAGTGAATGAGAAAGATAAGGAGT  TTTGAACCACTAACGAAAAAGGATAAATCAAAAGGATACGATAAATAATTAGGGAGTCAA  ATGGTCTTTTTGGGGATAGAGGGACTTGAACCCTCACGATTTTTGAAATCGACGGATTTT  CCTCTTACTATAAATTTCATTGTTGCCGGTATTGACATGTAGAACGGGACTCTATCTTTA  TTCTCGTCCAACAGTTCTTCAAAAGATCTATCAGATTATGGAGTGAATGGTTTGATCAAT  GGATATTCGATTCTTTCTTCAATATGGAATCGATTCATACCAATTCTTCTGTATTATGTA  TACAGGTTTATCCTTCAGCTTTTCTGAAGTTTCGATAGAAGGATTTCTCTACCAATGTAA  GACAATCAACTCCATTCGTTAGAACAACTTCCATCGAGTCTCTGCACCTATCCTTTTTTC  TTTTCGCTTTCTGAACCTTTGTTTGTTTTCATACAGGCGCGGCGATCAGTTGGACCTTTG  ATTAATTAACATCTCTTTTTTTG------ATTGACCTCCTCCTTTCTTTAATATCCAGGA  GGTCAAATTAAGATTGCTGTTCCAGTTAGTGTTTCAGTCGAATTCGATCGAAGAAGATCC  GAATCACGCTCTGTAGGATTTGAACCTACGACATCGGGTTTTGGAGACCCACGTTCTACC  GAACTGAACTAAGAGCGCTTTCTTATCATAAAAGATAAGATAAGACTGTAAAGAAAAGGA  TTGGCCCCAATACATCTTGTATGCATACACTATACAATATCGTAAGAATCGAAGATGTAA  GTTCTATATGATATGTCCAATGCGAATTGATCTCAAAAAATCCCTTGTTACTGCTCAAAG  GAGCAGTAATAAGTAGGGATGACAGGATTTGAACCCGTGACATTTTGTACCCAAAACAAA  CGCGCTACCAAGCCAGACATGCTGAGCTCCACATATTCTTGTACAGTCAAAAGGGGATCG  ATTCCGTAAAAGATTGGATCAGTAAATGGAAATTAACTGAAAAAATCTTTGTGAGATCGT  CAATATTGTACCGAGGGCGTCTTTAGAGTATACCGAATCAGTATAGCTATCCTTCTTCTG  ACACAGCAACGCACTTTCAATCAGTATTGGAAGGAGATGCTAAATAATTTATTTCTTCCT  TTACTTGTTGTTTGTCGATGTAAAATAATGTCGCATTCAATAGAAAATTTTAAAATTTTT  AAAAATCTATTTCTCGTTATTATGAGTTTAGGGCTAGAAACGGAGGATCAGGTAAAATCT  GAATCTGATAAGCAAGTTTCTAATAATTCATGAAATTTATTAGAATGCTCCCACAATTGT  AAGTAGATGTGAGATCTATAAATCTTTGTTATTCTATAAATCTTTGTTATTCATAGTTGT  AGAAGCACTTTTTGTTGGAATCTTTTTTT-CTTTTAAGGAATTGGTTAGTCGTCCAGTAA  CAAGTAAGAATAGTAAATTTTATTTTATAAACG------AAAAAGAACAAAGA-TCAAAC  AAATAATTGGAATCACTAATGCATGCACTGTTGTATTAAATCGAAATCTGAAGGTTCTTT  CTTGACCTAAAATGAAAGTAAGGGTTTTTTGTTCGCTGATAAAAAAAAGAAGAATTTTAG  AATTTGATTACATAATGATTCAAAAAGAGTTTTATTTTAAAATGAAATTACACGACCCAG  TTCTTCTTATTCGTTTATAAGTTGAGTTGAAAAATGATGCAAGAATGCATTCGCTGATTG  CAAACGCGGTATGCGTAGATGTTACAGATGATGAATCCATTTTATACAGTTGTGACTTTA  TCCTTGTTAGTGCTGTCTATAATGATATATGAATCAATTTAATTGGTATTTTTTTATCCA  ATTTTGCAAAAAAGGAAACTCAGGTAAGTGCTTTTT-ATAAACATATGTATAAAAAGAAC |
| H6 | AACATGCTTCTATGTCAATTGAAAGGGTATTACAAAGTCTCATGCAGGTCCTATAATTTG  TCAAAAGAACAACTTTGTAAGTTTCAGTCTGAATAAAAAAAATATCATTCAAATGGGGAT  TCCTTGCTCAAAGATGTTCATTTGTACATATATTATAT-TCCATTGTGATGTGATAACAG  AACA----TTCCGTTCAGATCCATTTGTATAAAGAGTAGTGAATGAGAAAGATAAGGAGT  TTTGAACCACTAACGAAAAAGGATAAATCAAAAGGATACGATAAATAATTAGGGAGTCAA  ATGGTCTTTTTGGGGATAGAGGGACTTGAACCCTCACGATTTTTGAAATCGACGGATTTT  CCTCTTACTATAAATTTCATTGTTGCCGGTATTGACATGTAGAACGGGACTCTATCTTTA  TTCTCGTCCAACAGTTCTTCAAAAGATCTATCAGATTATGGAGTGAATGGTTTGATCAAT  GGATATTCGATTCTTTCTTCAATATGGAATCGATTCATACCAATTCTTCTGTATTATGTA  TACAGGTTTATCCTTCAGATTTTCTGAAGTTTCGATAGAAGGATTTCTCTACCAATGTAA  GACAATCAACTCCATTCGTTAGAACAACTTCCATCGAGTCTCTGCACCTATCCTTTTTTC  TTTTCGCTTTCTGAACCTTTGTTTGTTTTCATACAGGCGCGGCGATCAGTTGGACCTTTG  ATTAATTAACATCTCTTTTTTTG------ATTGACCTCCTCCTTTCTTTAATATCCAGGA  GGTCAAATTAAGATTGATGTTCCAGTTAGTGTTTCAGTCGAATTCGATCGAAGAAGATCC  GAATCACGCTCTGTAGGATTTGAACCTACGACATCGGGTTTTGGAGACCCACGTTCTACC  GAACTGAACTAAGAGCGCTTTCTTATCATAAAAGATAAGATAAGACTGTAAAGAAAAGGA  TTGGCCCCAATACATCTTGTATGCATACACTATACAATATCGTAAGAATCGAAGATGTAA  GTTCTATATGATATGTCCAATGCGAATTGATCTCAAAAAATCCCTTGTTACTGCTCAAAG  GAGCAGTAATAAGTAGGGATGACAGGATTTGAACCCGTGACATTTTGTACCCAAAACAAA  CGCGCTACCAAGCCAGACATGCTGAGCTCCACATATTCTTGTACAGTCAAAAGGGGATCG  ATTCCGTAAAAGATTGGATCAGTAAATGGAAATTAACTGAAAAAATCTTTGTGAGATCGT  CAATATTGTACCGAGGGCGTCTTTAGAGTATACCGAATCAGTATAGCTATCCTTCTTCTG  ACACAGCAACGCACTTTCAATCAGTATTGGAAGGAGATGCTAAATAATTTATTTCTTCCT  TTACTTGTTGTTTGTCGATGTAAAATAATGTCGCATTCAATAGAAAATTTTAAAATTTTT  AAAAATATATTTCTCGTTATTATGAGTTTAGGGCTAGAAACGGAGGATCAGGTAAAATCT  GAATCTGATAAGCAAGTTTCTAATAATTCATGAAATTTATTAGAATGCTCCCACAATTGT  AAGTAGATGTGAGATCTATAAATCTTTGTTATTCTATAAATCTTTGTTATTCATAGTTGT  AGAAGCACTTTTTGTTGGAATCTTTTTTT-CTTTTAAGGAATTGGTTAGTCGTCCAGTAA  CAAGTAAGAATAGTAAATTTTATTTTATAAACG------AAAAAGAACAAAGA-TCAAAC  AAATAATTGGAATCACTAATGCATGCACTGTTGTATTAAATCGAAATCTGAAGGTTCTTT  CTTGACCTAAAATGAAAGTAAGGGTTTTTTGTTCGCTGATAAAAAAAAGAATAATTTTAG  AATTTGATTACATAATGATTCAAAAAGAGTTTTATTTTAAAATGAAATTACACGACCCAG  TTCTTCTTATTCGTTTATAAGTTGAGTTGAAAAATGATGCAAGAATGCATTCGCTGATTG  CAAACGCGGTATGCGTAGATGTTACAGATGATGAATCCATTTTATACAGTTGTGACTTTA  TCCTTGTTAGTGCTGTCTATAATGATATATGAATCAATTTAATTGGTATTTTTTTATCCA  ATTTTGCAAAAAAGGAAACTCAGGTAAGTGCTTTTT-ATAAACATATGTATAAAAAGAAC |
| H7 | AACATGCTTCTATGTCAATTGAAAGGGTATTACAAAGTCTCATGCAGGTCCTATAATTTG  TCAAAAGAACAACTTTGTAAGTTTCAGTCTGAATAAAAAAAATATCATTCAAATGGGGAT  TCCTTGCTCAAAGATGTTCATTTGTACATATATTATAT-TCCATTGTGATGTGATAACAG  AACA----TTCCGTTCAGATCCATTTGTATAAAGAGTAGTGAATGAGAAAGATAAGGAGT  TTTGAACCACTAACGAAAAAGGATAAATCAAAAGGATACGATAAATAATTAGGGAGTCAA  ATGGTCTTTTTGGGGATAGAGGGACTTGAACCCTCACGATTTTTGAAATCGACGGATTTT  CCTCTTACTATAAATTTCATTGTTGCCGGTATTGACATGTAGAACGGGACTCTATCTTTA  TTCTCGTCCAACAGTTCTTCAAAAGATCTATCAGATTATGGAGTGAATGGTTTGATCAAT  GGATATTCGATTCTTTCTTCAATATGGAATCGATTCATACCAATTCTTCTGTATTATGTA  TACAGGTTTATCCTTCAGATTTTCTGAAGTTTCGATAGAAGGATTTCTCTACCAATGTAA  GACAATCAACTCCATTCGTTAGAACAACTTCCATCGAGTCTCTGCACCTATCCTTTTTTC  TTTTCGCTTTCTGAACCTTTGTTTGTTTTCATACAGGCGCGGCGATCAGTTGGACCTTTG  ATTAATTAACATCTCTTTTTTTG------ATTGACCTCCTCCTTTCTTTAATATCCAGGA  GGTCAAATTAAGATTGATGTTCCAGTTAGTGTTTCAGTCGAATTCGATCGAAGAAGATCC  GAATCACGCTCTGTAGGATTTGAACCTACGACATCGGGTTTTGGAGACCCACGTTCTACC  GAACTGAACTAAGAGCGCTTTCTTATCATAAAAGATAAGATAAGACTGTAAAGAAAAGGA  TTGGCCCCAATACATCTTGTATGCATACACTATACAATATCGTAAGAATCGAAGATGTAA  GTTCTATATGATATGTCCAATGCGAATTGATCTCAAAAAATCCCTTGTTACTGCTCAAAG  GAGCAGTAATAAGTAGGGATGACAGGATTTGAACCCGTGACATTTTGTACCCAAAACAAA  CGCGCTACCAAGCCAGACATGCTGAGCTCCACATATTCTTGTACAGTCAAAAGGGGATCG  ATTCCGTAAAAGATTGGATCAGTAAATGGAAATTAACTGAAAAAATCTTTGTGAGATCGT  CAATATTGTACCGAGGGCGTCTTTAGAGTATACCGAATCAGTATAGCTATCCTTCTTCTG  ACACAGCAACGCACTTTCAATCAGTATTGGAAGGAGATGCTAAATAATTTATTTCTTCCT  TTACTTGTTGTTTGTCGATGTAAAATAATGTCGCATTCAATAGAAAATTTTAAAATTTTT  AAAAATATATTTCTCGTTATTATGAGTTTAGGGCTAGAAACGGAGGATCAGGTAAAATCT  GAATCTGATAAGCAAGTTTCTAATAATTCATGAAATTTATTAGAATGCTCCCACAATTGT  AAGTAGATGTGAGATCTATAAATCTTTGTTATTCTATAAATCTTTGTTATTCATAGTTGT  AGAAGCACTTTTTGTTGGAATCTTTTTTT-CTTTTAAGGAATTGGTTAGTCGTCCAGTAA  CAAGTAAGAATAGTAAATTTTATTTTATAAACG------AAAAAGAACAAAGA-TCAAAC  AAATAATTGGAATCACTAATGCATGCACTGTTGTATTAAATCGAAATCTGAAGGTTCTTT  CTTGACCTAAAATGAAAGTAAGGGTTTTTTGTTCGCTGATAAAAAAAAGAAGAATTTTAG  AATTTGATTACATAATGATTCAAAAAGAGTTTTATTTTAAAATGAAATTACACGACCCAG  TTCTTCTTATTCGTTTATAAGTTGAGTTGAAAAATGATGCAAGAATGCATTCGCTGATTG  CAAACGCGGTATGCGTAGATGTTACAGATGATGAATCCATTTTATACAGTTGTGACTTTA  TCCTTGTTAGTGCTGTCTATAATGATATATGAATCAATTTAATTGGTATTTTTTTATCCA  ATTTTGCAAAAAAGGAAACTCAGGTAAGTGCTTTTT-ATAAACATATGTATAAAAAGAAC |
| H8 | AACATGCTTCTATGTCAATTGAAAGGGTATTACAAAGTCTCATGCAGGTCCTATAATTTG  TCAAAAGAACAACTTTGTAAGTTTCAGTCTGAATAAAAAAAATATCATTCAAATGGGGAT  TCCTTGCTCAAAGATGTTCATTTGTACATATATTATAT-TCCATTGTGATGTGATAACAG  AACA----TTCCGTTCAGATCCATTTGTATAAAGAGTAGTGAATGAGAAAGATAAGGAGT  TTTGAACCACTAACGAAAAAGGATAAATCAAAAGGATACGATAAATAATTAGGGAGTCAA  ATGGTCTTTTTGGGGATAGAGGGACTTGAACCCTCACGATTTTTGAAATCGACGGATTTT  CCTCTTACTATAAATTTCATTGTTGCCGGTATTGACATGTAGAACGGGACTCTATCTTTA  TTCTCGTCCAACAGTTCTTCAAAAGATCTATCAGATTATGGAGTGAATGGTTTGATCAAT  GGATATTCGATTCTTTCTTCAATATGGAATCGATTCATACCAATTCTTCTGTATTATGTA  TACAGGTTTATCCTTCAGATTTTCTGAAGTTTCGATAGAAGGATTTCTCTACCAATGTAA  GACAATCAACTCCATTCGTTAGAACAACTTCCATCGAGTCTCTGCACCTATCCTTTTTTC  TTTTCGCTTTCTGAACCTTTGTTTGTTTTCATACAGGCGCGGCGATCAGTTGGACCTTTG  ATTAATTAACATCTCTTTTTTTG------ATTGACCTCCTCCTTTCTTTAATATCCAGGA  GGTCAAATTAAGATTGATGTTCCAGTTAGTGTTTCAGTCGAATTCGATCGAAGAAGATCC  GAATCACGCTCTGTAGGATTTGAACCTACGACATCGGGTTTTGGAGACCCACGTTCTACC  GAACTGAACTAAGAGCGCTTTCTTATCATAAAAGATAAGATAAGACTGTAAAGAAAAGGA  TTGGCCCCAATACATCTTGTATGCATACACTATACAATATCGTAAGAATCGAAGATGTAA  GTTCTATATGATATGTCCAATGCGAATTGATCTCAAAAAATCCCTTGTTACTGCTCAAAG  GAGCAGTAATAAGTAGGGATGACAGGATTTGAACCCGTGACATTTTGTACCCAAAACAAA  CGCGCTACCAAGCCAGACATGCTGAGCTCCACATATTCTTGTACAGTCAAAAGGGGATCG  ATTCCGTAAAAGATTGGATCAGTAAATGGAAATTAACTGAAAAAATCTTTGTGAGATCGT  CAATATTGTACCGAGGGCGTCTTTAGAGTATACCGAATCAGTATAGCTATCCTTCTTCTG  ACACAGCAACGCACTTTCAATCAGTATTGGAAGGAGATGCTAAATAATTTATTTCTTCCT  TTACTTGTTGTTTGTCGATGTAAAATAATGTCGCATTCAATAGAAAATTTTAAAATTTTT  AAAAATATATTTCTCGTTATTATGAGTTTAGGGCTAGAAACGGAGGATCAGGTAAAATCT  GAATCTGATAAGCAAGTTTCTAATAATTCATGAAATTTATTAGAATGCTCCCACAATTGT  AAGTAGATGTGAGATCTATAAATCTTTGTTATTCTATAAATCTTTGTTATTCATAGTTGT  AGAAGCACTTTTTGTTGGAATCTTTTTTT-CTTTTAAGGAATTGGTTAGTCGTCCAGTAA  CAAGTAAGAATAGTCAATTTTATTTTATAAACG------AAAAAGAACAAAGA-TCAAAC  AAATAATTGGAATCACTAATGCATGCACTGTTGTATTAAATCGAAATCTGAAGGTTCTTT  CTTGACCTAAAATGAAAGTAAGGGTTTTTTGTTCGCTGATAAAAAAAAGAAGAATTTTAG  AATTTGATTACATAATGATTCAAAAAGAGTTTTATTTTAAAATGAAATTACACGACCCAG  TTCTTCTTATTCGTTTATAAGTTGAGTTGAAAAATGATGCAAGAATGCATTCGCTGATTG  CAAACGCGGTATGCGTAGATGTTACAGATGATGAATCCATTTTATACAGTTGTGACTTTA  TCCTTGTTAGTGCTGTCTATAATGATATATGAATCAATTTAATTGGTATTTTTTTATCCA  ATTTTGCAAAAAAGGAAACTCAGGTAAGTGCTTTTT-ATAAACATATGTATAAAAAGAAC |
| H9 | AACATGCTTCTATGTCAATTGAAAGGGTATTACAAAGTCTCATGCAGGTCCTATAATTTG  TCAAAAGAACAACTTTGTAAGTTTCAGTCTGAATAAAAAAAATATCATTCAAATGGGGAT  TCCTTGCTCAAAGATGTTCATTTGTACATATATTATAT-TCCATTGTGATGTGATAACAG  AACA----TTCCGTTCAGATCCATTTGTATAAAGAGTAGTGAATGAGAAAGATAAGGAGT  TTTGAACCACTAACGAAAAAGGATAAATCAAAAGGATACGATAAATAATTAGGGAGTCAA  ATGGTCTTTTTGGGGATAGAGGGACTTGAACCCTCACGATTTTTGAAATCGACGGATTTT  CCTCTTACTATAAATTTCATTGTTGCCGGTATTGACATGTAGAACGGGACTCTATCTTTA  TTCTCGTCCAACAGTTCTTCAAAAGATCTATCAGATTATGGAGTGAATGGTTTGATCAAT  GGATATTCGATTCTTTCTTCAATATGGAATCGATTCATACCAATTCTTCTGTATTATGTA  TACAGGTTTATCCTTCAGATTTTCTGAAGTTTCGATAGAAGGATTTCTCTACCAATGTAA  GACAATCAACTCCATTCGTTAGAACAACTTCCATCGAGTCTCTGCACCTATCCTTTTTTC  TTTTCGCTTTCTGAACCTTTGTTTGTTTTCATACAGGCGCGGCGATCAGTTGGACCTTTG  ATTAATTAACATCTCTTTTTTTG------ATTGACCTCCTCCTTTCTTTAATATCCAGGA  GGTCAAATTAAGATTGATGTTCCAGTTAGTGTTTCAGTCGAATTCGATCGAAGAAGATCC  GAATCACGCTCTGTAGGATTTGAACCTACGACATCGGGTTTTGGAGACCCACGTTCTACC  GAACTGAACTAAGAGCGCTTTCTTATCATAAAAGATAAGATAAGACTGTAAAGAAAAGGA  TTGGCCCCAATACATCTTGTATGCATACACTATACAATATCGTAAGAATCGAAGATGTAA  GTTCTATATGATATGTCCAATGCGAATTGATCTCAAAAAATCCCTTGTTACTGCTCAAAG  GAGCAGTAATAAGTAGGGATGACAGGATTTGAACCCGTGACATTTTGTACCCAAAACAAA  CGCGCTACCAAGCCAGACATGCTGAGCTCCACATATTCTTGTACAGTCAAAAGGGGATCG  ATTCCGTAAAAGATTGGATCAGTAAATGGAAATTAACTGAAAAAATCTTTGTGAGATCGT  CAATATTGTACCGAGGGCGTCTTTAGAGTATACCGAATCAGTATAGCTATCCTTCTTCTG  ACACAGCAACGCACTTTCAATCAGTATTGGAAGGAGATGCTAAATAATTTATTTCTTCCT  TTACTTGTTGTTTGTCGATGTAAAATAATGTCGCATTCAATAGAAAATTTGAAAATTTTT  AAAAATATATTTCTCGTTATTATGAGTTTAGGGCTAGAAACGGAGGATCAGGTAAAATCT  GAATCTGATAAGCAAGTTTCTAATAATTCATGAAATTTATTAGAATGCTCCCACAATTGT  AAGTAGATGTGAGATCTATAAATCTTTGTTATTCTATAAATCTTTGTTATTCATAGTTGT  AGAAGCACTTTTTGTTGGAATCTTTTTTT-CTTTTAAGGAATTGGTTAGTCGTCCAGTAA  CAAGTAAGAATAGTCAATTTTATTTTATAAACG------AAAAAGAACAAAGA-TCAAAC  AAATAATTGGAATCACTAATGCATGCACTGTTGTATTAAATCGAAATCTGAAGGTTCTTT  CTTGACCTAAAATGAAAGTAAGGGTTTTTTGTTCGCTGATAAAAAAAAGAAGAATTTTAG  AATTTGATTACATAATGATTCAAAAAGAGTTTTATTTTAAAATGAAATTACACGACCCAG  TTCTTCTTATTCGTTTATAAGTTGAGTTGAAAAATGATGCAAGAATGCATTCGCTGATTG  CAAACGCGGTATGCGTAGATGTTACAGATGATGAATCCATTTTATACAGTTGTGACTTTA  TCCTTGTTAGTGCTGTCTATAATGATATATGAATCAATTTAATTGGTATTTTTTTATCCA  ATTTTGCAAAAAAGGAAACTCAGGTAAGTGCTTTTT-ATAAACATATGTATAAAAAGAAC |
