# Supplementary material for: Population Structure, Genetic Diversity, and Evolutionary History of Kleinia neriifolia (Asteraceae) on the Canary Islands
Source: Front Plant Sci. 2017 Jun 30;8:1180. doi: 10.3389/fpls.2017.01180 (PMC5492869; doi:10.3389/fpls.2017.01180)
Supplement: Supplementary file 3 [file Table_3.DOCX]

Table S3. Prior distribution for each parameter used in each scenario. The first eight are time parameters (measured in generation units), and the other eight are population effective size (measured in number of individuals). In all cases the minimum and maximum values are shown. Names of each population and periods of time correspond to those of Figure 1.

| Parameter | Minimum value | Maximum value | Units of each parameter |
| --- | --- | --- | --- |
| *t1* | 1 | 1,000 | Generations |
| *t2* | 1,000 | 5,000 | Generations |
| *t3* | 5,000 | 10,000 | Generations |
| *t4* | 10,000 | 50,000 | Generations |
| *t5* | 50,000 | 100,000 | Generations |
| *t6* | 100,000 | 300,000 | Generations |
| *t7* | 300,000 | 500,000 | Generations |
| *t8* | 500,000 | 1,000,000 | Generations |
| *NA* | 10 | 100,000 | Individuals |
| *N1* | 10 | 100,000 | Individuals |
| *N2* | 10 | 100,000 | Individuals |
| *N3* | 10 | 100,000 | Individuals |
| *N4* | 10 | 100,000 | Individuals |
| *N5* | 10 | 100,000 | Individuals |
| *N6* | 10 | 100,000 | Individuals |
| *N7* | 10 | 100,000 | Individuals |
