# Supplementary material for: Population Structure, Genetic Diversity, and Evolutionary History of Kleinia neriifolia (Asteraceae) on the Canary Islands
Source: Front Plant Sci. 2017 Jun 30;8:1180. doi: 10.3389/fpls.2017.01180 (PMC5492869; doi:10.3389/fpls.2017.01180)
Supplement: Supplementary file 4 [file Table_4.DOCX]

Table S4. Values of each parameter estimated in Scenario 6. Mean, Median and 95% confidence interval are shown (q025 lower and q975 upper limits). Names of each population and periods of time correspond to those of Figure 1.

| Parameter | Mean | Median | q025 | q975 |
| --- | --- | --- | --- | --- |
| *N1* | 4.30E+04 | 3.84E+04 | 9.85E+03 | 9.37E+04 |
| *N2* | 2.31E+04 | 1.89E+04 | 5.62E+03 | 7.04E+04 |
| *N3* | 2.81E+04 | 2.43E+04 | 7.45E+03 | 7.37E+04 |
| *N4* | 5.13E+04 | 5.02E+04 | 2.06E+04 | 9.02E+04 |
| *N5* | 4.80E+04 | 4.67E+04 | 2.03E+04 | 8.65E+04 |
| *N6* | 2.98E+04 | 2.34E+04 | 4.99E+03 | 8.64E+04 |
| *N7* | 2.52E+04 | 1.69E+04 | 2.93E+03 | 8.73E+04 |
| *NA* | 3.60E+04 | 2.96E+04 | 1.06E+03 | 9.36E+04 |
| *t1* | 8.71E+02 | 9.32E+02 | 3.64E+02 | 9.98E+02 |
| *t2* | 4.20E+03 | 4.47E+03 | 1.94E+03 | 4.98E+03 |
| *t3* | 6.95E+03 | 6.73E+03 | 5.07E+03 | 9.71E+03 |
| *t4* | 1.38E+04 | 1.18E+04 | 1.01E+04 | 3.12E+04 |
| *t5* | 6.25E+04 | 5.83E+04 | 5.03E+04 | 9.36E+04 |
| *t8* | 2.05E+05 | 2.08E+05 | 1.07E+05 | 2.95E+05 |
